# The Use of Self-Expanding Plastic Stents in the Management of Oesophageal Leaks and Spontaneous Oesophageal Perforations

**DOI:** 10.1155/2011/418103

**Published:** 2011-07-07

**Authors:** L. H. Moyes, C. K. MacKay, M. J. Forshaw

**Affiliations:** Department of Surgery, Oesophagogastric Unit, Royal Infirmary, Glasgow G4 0SF, UK

## Abstract

Leakage after oesophageal anastomosis or perforation remains a challenge for the surgeon. Traditional management has been operative repair or intensive conservative management. Both treatments are associated with prolonged hospitalisation and high morbidity and mortality rates. Self-expanding metallic stents have played an important role in the palliation of malignant oesophageal strictures and the treatment of tracheoesophageal fistulae. However, self-expanding metal stents in benign oesophageal disease are associated with complications such as bleeding, food bolus impaction, stent migration, and difficulty in retrieval. The Polyflex stent is the only commercially available self-expanding plastic stent which has been used in the management of malignant oesophageal strictures with good results. This review will consider the literature concerning the use of self-expanding plastic stents in the treatment of oesophageal anastomotic leakage and spontaneous perforations of the oesophagus.

## 1. Introduction

Stents have played an important role over the last few years for the palliation of malignant oesophageal strictures and the treatment of tracheoesophageal fistulae [1]. There are several types of commercial stents available, but they can largely be separated into two groups: metal or plastic stents. Self-expanding metal stents (SEMSs) are made of stainless steel (Z stent, Cook), nitinol (Ultraflex, Boston Scientific), or elgiloy (Wallstent, Boston Scientific) and may be uncovered, partially covered, or fully covered. Uncovered or partially covered stents (e.g., Ultraflex) allow fixing to the oesophageal lumen, but they have a tendency to become blocked due to tumour overgrowth. Fully covered metal stents (e.g., Niti-S) are beneficial in controlling leakage and can be easily retrieved, but they are prone to migration. There is currently only one commercially available plastic stent on the market—the Polyflex stent produced by Boston Scientific. These stents have been successfully used in the management of benign and malignant oesophageal strictures [2].

SEMS replaced rigid metal stents in the 1990s and have been successfully used for the management of tracheoesophageal fistulae and malignant strictures [3, 4]. They are relatively easy to deploy, have a high success rate, and provide rapid symptom relief. There are also reports in the literature regarding the use of SEMS for the successful treatment of benign oesophageal disease including perforations. However, these partially covered or uncovered SEMSs were associated with relatively high complication rates (26–52%) including bleeding, perforation, stent migration, pain, ingrowth, and food bolus impaction [5]. As a result, many centres would not advocate the use of uncovered or partially covered stents in benign disease [6].

The Polyflex stent is a self-expanding plastic stent (SEPS) of polyester braid completely covered in silicone membrane. The proximal end is flared to prevent dislocation and ensure reliable occlusion with radioopaque markers at both ends and in the middle to facilitate accurate placement. The Polyflex stent overcomes some of the disadvantages associated with SEMS allowing easier retrieval and possibly less migration than uncovered or partially covered SEMS [7]. The soft material ensures well-balanced radial force and adapts to the oesophageal wall allowing reliable leak occlusion while the complete silicone covering prevents ingrowth and overgrowth of granulation tissue making it easier to reposition and retrieve. This can be done endoscopically with specially designed forceps. The Polyflex stent is available in various sizes with diameters of 16, 18, and 21 mm and lengths of 9, 12, or 15 cm. The exact size of stent will depend on the site and the size of the leakage, the operator's preference and the size of the oesophagus. A study assessing stent size and migration rates using SEMS in patients with malignant dysphagia suggests that a larger-diameter stent is associated with reduced migration rates. However, as yet there is no published evidence that this applies to SEPS [8]. 

Recently, there has been a move towards managing oesophageal leakage either secondary to perforations (spontaneous or iatrogenic) or from oesophageal anastomoses (after oesophagectomy or total gastrectomy), in a nonoperative way with the use of nutritional support, pleural/mediastinal drainage, and broad spectrum antibiotics in a critical care setting. There are now reports in the literature using the Polyflex stent, avoiding the morbidity and mortality of further surgical procedures. 

This paper will consider the literature concerning the use of self-expanding plastic stents in the treatment of oesophageal anastomotic leakage and spontaneous perforations of the oesophagus.

## 2. Literature Search

A literature search was performed using PubMed, Medline, and Embase databases searching for the English literature (reviews, original articles and case reports) available since 1975. The search was performed with mesh terms “oesophageal anastomotic leak”, “Polyflex stents”, “SEMS”, “SEPS” and “spontaneous oesophageal rupture”. All related articles were examined.

## 3. Anastomotic Leaks

Oesophagectomy and total gastrectomy remain challenging operations that are associated with considerable morbidity and mortality even in specialised centres. The National Oesophagogastric Cancer Audit of England and Wales published anastomotic leak rates of 8.3% after oesophagectomy and 5.9% after gastrectomy [9]. Despite improvements in anastomotic technique and perioperative care, intrathoracic anastomotic leakage is associated with contamination, large abscesses, and fistulas into pleural cavities which are often difficult to control. The constant leakage of gastric juices and saliva into the pleural and mediastinal cavities make this a life-threatening condition responsible for 30–40% of postoperative deaths [10–12].

The management of oesophagogastric and oesophagojejunal anastomotic leakage remains controversial. Some authors recommend aggressive surgical treatment, whereas others advocate a conservative approach of perianastomotic drainage, parenteral nutritional support, nasogastric decompression, and intravenous broad-spectrum antibiotics. These patients should all be managed in an appropriate critical care environment [11, 12]. Patients in both groups have considerable mortality rates and prolonged ICU and hospital stays.

The application of fibrin glue and endoclips have been successfully used in the management of very small oesophageal leaks [13, 14]. An endoscopic treatment would seem an attractive option, and the Polyflex stent offers promising results. 

Table 1 summarises the relevant case series with the indications, complications, and outcomes [7, 15–19]. These data suggest that plastic stents seem to be straightforward to place with an almost 100% immediate placement success. In our personal experience, the loading device and subsequent delivery of the stent can be challenging and associated with a significant learning curve. A further difficulty is that the diameter of the stent delivery device is 12–14 mm, and in some situations, dilation prior to stent insertion may be required. However, this is more likely to be encountered in cases of stricturing disease rather than leakage. The immediate leak occlusion rates varied from 60–100% with more than 90% healing rates. Leak occlusion was assessed by water soluble contrast studies and endoscopic assessment. Stents were removed at various points, but the majority were removed between 14 and 28 days with healing assessed clinically by the absence of sepsis and reduction in chest drain effluent, endoscopically and radiologically by contrast studies. While the literature is limited regarding optimal timing of stent placement, the majority of series-favoured stent placement immediately after the diagnosis was made in order to minimise or control contamination into mediastinal or pleural cavities [15]. However, even delayed placement resulted in closure of the anastomotic leak. Patients treated with Polyflex stent had earlier oral intake (mean 11 days versus 23 days), less extensive ICU stay (mean 25 days versus 47 days), and a shorter overall hospital stay (mean 35 days versus 57 days). The inhospital mortality rate across the series varied from 0–20% which was lower than that in the conservatively treated arm [16, 18].

## 4. Spontaneous Oesophageal Perforation

Spontaneous oesophageal perforation is a life-threatening condition that traditionally requires surgical repair. Management of this condition can be divided into two categories—conservative and operative. Due to the uncommon nature of this condition, the literature is based mainly on small case series and individual reports. 

Operative intervention still seems to be the best treatment for cases with an early diagnosis. Reported mortality rates vary from 0% if treatment started within 24 hours to 30% if delayed [20]. 

Conservative treatment consists of broad-spectrum antibiotic therapy, nutritional support, and/or percutaneous drainage of collections. Ivey and colleagues suggest conservative therapy is only appropriate if the following criteria are met—the perforation is five days old, there are no signs of severe sepsis, there is a wide mouth cavity on contrast studies draining freely back into the oesophagus, and the pleural space is not contaminated [21]. A recent review suggests that conservative measures seem feasible with survival rates of 60–70% in selected cases if patients are diagnosed promptly and are not septic, but conversion to surgery remains necessary in those who fail to progress [22]. 

Griffin et al. published their experience of the management of spontaneous oesophageal perforation and do not advocate the use of stents in this situation. They feel the stent may prevent adequate drainage of sepsis, delay healing, and be subject to migration as there is no stricture to keep it in place. They would recommend intensive nonoperative management in carefully selected patients with a low threshold for surgery [20].

There are only three case reports in the literature describing the successful treatment of spontaneous rupture with temporary placement of a SEPS (Polyflex stent). One was placed in the immediate period (within 24 hrs), another in a delayed (three weeks) setting after a trial of conservative management with chest drainage, antibiotics, and fibrin glue, and the final case placed ten days after initial treatment. All patients survived, and immediate postprocedure studies showed no extravasation of contrast [15, 23, 24]. Full oral intake was resumed in all patients within one week, and all patients discharged between 7 and 21 days postprocedure. Elective stent removal was carried out between 5 and 10 weeks. In two cases, the stent had migrated into the stomach. All patients were well at 6 months with no difficulty in swallowing.

## 5. Discussion

The Polyflex self-expanding plastic stent appears to be a feasible alternative option in the treatment of patients with anastomotic leaks following oesophagectomy, total gastrectomy, and spontaneous oesophageal perforation. Surgery for both conditions is associated with significant morbidity and mortality even in specialised centres, but the use of endoscopic therapies may improve outcomes in selected patients.

The Polyflex stent itself is relatively simple to place endoscopically, comes in a range of sizes, is effective, and can be easily repositioned and removed. Patients with intrathoracic anastomotic leakage and spontaneous oesophageal perforation are often critically ill, and the option of endoscopic intervention without the stresses associated with major surgery must be an attractive option. While the literature surrounding the use of Polyflex stents is still in its infancy, the results are interesting and encouraging.

The general advantage of the Polyflex stent is immediate occlusion of the anastomotic leakage or perforation at one endoscopic session that allows earlier oral/enteral feeding. Patients treated with the Polyflex stent had earlier oral intake, less extensive ICU course, and shorter hospital stays [16]. The Polyflex stent allows nonoperative treatment of patients with anastomotic leak who historically would have required surgical intervention [25]. The plastic stent overcomes the difficulties of the well-recognised complications associated with partially covered SEMS—perforation, bleeding, and difficulty in retrieval—because their uncovered ends quickly become embedded in the oesophageal wall. In some patients, argon plasma coagulation is required to remove the stent, and post procedure stenoses from extensive mucosal damage are documented [26–28].

The main disadvantage throughout the literature is the tendency of the Polyflex stent to migrate with reported rates between 5 and 23% (see Table 1). Its complete silicone coating allows easy retrieval, but it is this property that makes it prone to migration as there is minimal granulation reaction. Some groups have tried to keep the stent position using endoclips at the proximal and distal ends with limited success. Dai et al. reported reintervention rates of 80% (4 of 5 patients with stent migration), although these were successfully treated with repeat endoscopic intervention, either by repositioning the stent with forceps or clips, or to be restenting [7]. The other concern suggested by Griffin is that stents may prevent adequate drainage in cases with spontaneous oesophageal perforation. Spontaneous perforation of the oesophagus is associated with significant contamination of the pleural and mediastinal cavities and the presence of a covered stent, although controlling the leakage may result in inadequate drainage [20]. They recommend intensive nonoperative management with a low threshold for surgery. 

Most authors suggest carrying out stent placement immediately after the diagnosis is made to limit further contamination. Although there is no evidence from SEPS about the timing of stent placement and healing, there is indirect evidence from endoscopic clipping in the management of oesophageal perforations. Qadeer et al. describe 17 patients in whom endoscopic clipping was used to close oesophageal perforations (mainly iatrogenic) and showed that the median healing time after clipping was 18 days [14]. Most of the acute perforations from therapeutic endoscopy closed by 5 days, but in older perforations initially treated conservatively before undergoing endoscopic clipping, there was a delay in healing time. For every 10-day increase in the duration of the perforation, healing time increased by 7 days. All patients should be managed in a critical care setting, with broad-spectrum antibiotic therapy, nutritional support, and effective drainage of the perianastomotic area and mediastinal/pleural cavities to avoid septic complications. This may be achieved from chest drainage tubes if they are still in place, or the insertion of CT-guided percutaneous catheters. Some authors feel a dehiscence of more than 70% of the anastomosis with an ischaemic anastomotic line tends not suitable for endoscopic treatment, and reoperation is required [17, 18]. If clinical markers or physiological parameters are not improving, reoperation should be considered. There is discrepancy in the literature concerning the retrieval of the Polyflex stent once the leak has healed. Some groups remove the stent at 14 days and assess healing and viability of the oesophagus [18], while some groups favour stent removal only when it becomes troublesome [17]. Further studies are required before final conclusions are drawn. The published literature commenting on stent size in patients with oesophageal leakage is poor, although common sense would dictate that the larger stent is required in this situation due to the lack of oesophageal stricture.

The main principles from the literature suggest that all patients regardless of the underlying pathology should be managed in a critical care setting, with broad spectrum antibiotic therapy, nutritional support, and adequate drainage of the pleural and mediastinal cavities to avoid septic complications. Appropriate drainage of cavities is essential, particularly in the case of spontaneous oesophageal perforation where food debris can lie in the pleural or mediastinal cavities resulting in significant septic complications. These cavities must be drained by well-placed surgical or radiologically guided drains, but in some cases, a minithoracotomy (either surgical or laparoscopic assisted) and washout may be required to control contamination. The management of these complex patients should involve a multidisciplinary team comprising a surgeon, a competent endoscopist, and an interventional radiologist. Nonoperative management with stents should be instituted as quickly as possible to minimise pleural and mediastinal contamination. Surgical reexploration, however, should always be considered in patients who do not clinically improve with this treatment. 

The literature suggests that plastic stents may be of use in patients with leakage from an oesophageal anastomosis but perhaps not for spontaneous perforation. If the dehiscence is more than 70% and ischaemia/tension present, most groups would be advocated reoperation, with the stents being used in smaller leaks and patients who may not survive further surgery [18].

Two fully covered retrievable SEMS, the Niti-S (TaeWoong) and the covered Wallflex (Boston Scientific), have recently been FDA approved for the use in malignant oesophageal strictures although there is no data to support their routine use in benign disease including perforations at present [2]. A study assessing the efficacy of the alveolus oesophageal stent system (a nitonol stent fully covered internally allowing the outer portion to adhere to the oesophageal lumen) has be shown to successfully occlude the leak or fistula in 4 of 7 (57%) patients [29]. The Polyflex stent and newer fully covered metal stents are attractive options for the use in controlling oesophageal leakage and may be more cost effective than currently used partially covered SEMS. However, further research in this area is required to assess the long-term clinical and financial implications.

A further area of interest is the use of biodegradable stents in the management of benign oesophageal conditions as this would negate the need for stent removal [2]. Although these are exciting prospects, long-term data from good-quality studies needs to be collated before these stents are widely adopted into clinical practice. However, while these stents may have a role in the management of benign stricture disease, their use in oesophageal perforation seems unlikely as they are not covered and would not control leakage.

## 6. Conclusion

The literature search has shown that data is scarce, and the paucity of large case series or trials has resulted in a lack of clinical standards or guidelines to aid management of patients with oesophageal leakage. However, the limited results are promising, and endoscopic therapy with a self-expanding stent should be borne in mind when treating patients with oesophageal leakage.

## Figures and Tables

**Table 1:** Summary of the literature regarding Polyflex stents and anastomotic leakage.

Reference	Study	Patients	Leak site	Stent	Timing after diagnosis	Placement success	Immediate leak occlusion	% healing	Complications	Outcome
Gelbmann [15]	Case series	5	OG anastomosis	Plastic	13–65 days (too unwell for surgery)	100%	60% 40% ↓ leakage	80%		20% mortality (multiorgan failure) 10% further stent
Hunerbein [16]	Stent versus conservative	199 stent 10 no stent	OG anastomosis	Plastic	Immediate (2-3 days)	100%	90%	100%	None	Stent group: earlier oral intake, shorter hospital stay, and 0% mortality
Langer [17]	Case series	20	OG and OJ anastomosis	Plastic	Immediate	90%	85%	90%	Stent misplacement in 10% increased dehiscence and required surgery	Stent migration and dislocation 10%
Schubert [18]	Case series	12	OG anastomosis	Plastic	Immediate	100%	92%	92%	Stent migration 17%	All healed One persistent leak closed with endoclip
Repici [19]	Case report	1	OJ anastomosis	Plastic	Delayed—persistent fistula (39 days)	100%	100%	100%	None	Stent removed 3 weeks Well
Dai [7]	Case series	22	OG anastomosis	Plastic	Immediate (2.5 days)	100%	95%	95%	23% migration	5% thoracotomy for persistent leak 5% mortality 5% dilatation of anastomotic stricture
